# Activating Akt1 mutations alter DNA double strand break repair and radiosensitivity

**DOI:** 10.1038/srep42700

**Published:** 2017-02-17

**Authors:** S. Oeck, K. Al-Refae, H. Riffkin, G. Wiel, R. Handrick, D. Klein, G. Iliakis, V. Jendrossek

**Affiliations:** 1 Institute of Cell Biology (Cancer Research), University of Duisburg-Essen, Medical School, Virchowstrasse 173, 45122 Essen, Germany; 2 Institute of Applied Biotechnology, University of Applied Sciences Biberach, Hubertus-Liebrecht-Str. 35, 88400 Biberach, Germany; 3 Institute of Medical Radiation Biology, University of Duisburg-Essen, Medical School, Hufelandstr. 55, 45122 Essen, Germany

## Abstract

The survival kinase Akt has clinical relevance to radioresistance. However, its contributions to the DNA damage response, DNA double strand break (DSB) repair and apoptosis remain poorly defined and often contradictory. We used a genetic approach to explore the consequences of genetic alterations of Akt1 for the cellular radiation response. While two activation-associated mutants with prominent nuclear access, the phospho-mimicking Akt1-TDSD and the clinically relevant PH-domain mutation Akt1-E17K, accelerated DSB repair and improved survival of irradiated Tramp-C1 murine prostate cancer cells and Akt1-knockout murine embryonic fibroblasts *in vitro*, the classical constitutively active membrane-targeted myrAkt1 mutant had the opposite effects. Interestingly, DNA-PKcs directly phosphorylated Akt1 at S473 in an *in vitro* kinase assay but not vice-versa. Pharmacological inhibition of DNA-PKcs or Akt restored radiosensitivity in tumour cells expressing Akt1-E17K or Akt1-TDSD. In conclusion, Akt1-mediated radioresistance depends on its activation state and nuclear localization and is accessible to pharmacologic inhibition.

Many human tumours harbour mutations leading to the hyperactivation of Akt (Protein Kinase B). These include the oncogenic activation of receptor tyrosine kinases, RAS or phosphatidylinositol-3-kinase (PI3K), loss of the tumour suppressor Phosphatase and Tensin Homolog (PTEN), or genomic amplification or gain-of-function mutations in one of the three Akt isoforms (Akt1, 2, 3)^1^,^2^,^3^,^4^. As a consequence, components of the PI3K/Akt signalling network attracted major attention for targeted anticancer drug development^5^,^6^. To date, PI3K pathway inhibitors are increasingly used in cancer treatment as single drugs or combined with radiotherapy and chemotherapy^7^,^8^,^9^.

Generally, reversible phosphorylation regulates Akt-activation at Threonine-308 (T308) and Serine-473 (S473). Furthermore, its activity is modulated by dephosphorylation, ubiquitination as well as environmental signals, e.g. availability of nutrients, growth factors or oxygen^1^,^9^,^10^,^11^,^12^. Akt influences almost all aspects of tumour biology and enhances the resistance of cancer (stem) cells to genotoxic stress^1^,^13^. In addition, the evidence is increasing for an intricate link between Akt and the regulation of DNA double strand break (DSB) repair through DNA-PK-dependent non-homologous end joining (D-NHEJ) and/or homologous recombination repair (HRR)^14^,^15^,^16^,^17^,^18^,^19^,^20^. Consequently, Akt-dependent DSB repair may give tumour cells intrinsic therapy resistance^19^. Yet, the role of Akt in DSB repair is still highly controversial: While Akt inhibition decreased DNA-PKcs-dependent DSB repair and increased the cytotoxicity of chemotherapy and ionizing radiation in preclinical investigations^16^,^20^,^21^,^22^,^23^, elevated Akt activity unexpectedly also reduced D-NHEJ efficiency at least in PTEN-deficient cancer cells, presumably by inhibiting XRCC4-like factor (XLF)^17^. In contrast to this, genomic amplification of Akt3 activated DNA DSB repair^4^. However, the consequences of mutations of Akt for the cellular radiation response have not yet been investigated.

Here, we used a genetic approach to systematically explore the mechanisms by which Akt impacts on DNA DSB repair and the cell fate after exposure to ionizing radiation, as well as to unravel a possible crosstalk with DNA-PKcs. In addition to the classical inactive kinase model (K179A) and constitutively active (myrAkt1) variant, we generated a phospho-mimicking (TDSD) as well as a pleckstrin homology (PH) domain mutant (R25C) with reduced membrane recruitment to extend the set of mutants with artificially increased or decreased Akt activity^24^. Moreover, we performed *in silico* research in the publically available COSMIC database to explore the occurrence and the frequency of somatic mutations in the three Akt isoforms with clinical relevance to cancer patients. As a consequence, we included a gain-of-function Akt1 mutation (c.49 G > A) in our study. This mutation results in a glutamic acid to lysine substitution at amino acid 17 (E17K) in the binding pocket of the PH domain of Akt1, thereby increasing PIP_3_-mediated recruitment to the cell membrane and affecting the response to the inhibition of Akt1’s kinase activity^3^,^25^. The E17K mutation turned out to be the only Akt mutation occurring with a substantial frequency in tumour samples of cancer patients. The E17K mutation is mostly mutually exclusive with other PI3K/Akt pathway activating alterations^26^,^27^ and occurs at low frequency in several human cancers that are frequently treated with radiotherapy, such as tumours of the breast, intestines, lung, and prostate^3^,^26^,^28^. Since the frequency of mutations including E17K in cancer patients was highest in Akt1, we focused on Akt1 in our genetic study.

## Results

### Akt1 is the dominant Akt isoform harbouring E17K mutations in the pleckstrin homology domain

**Figure 1:**
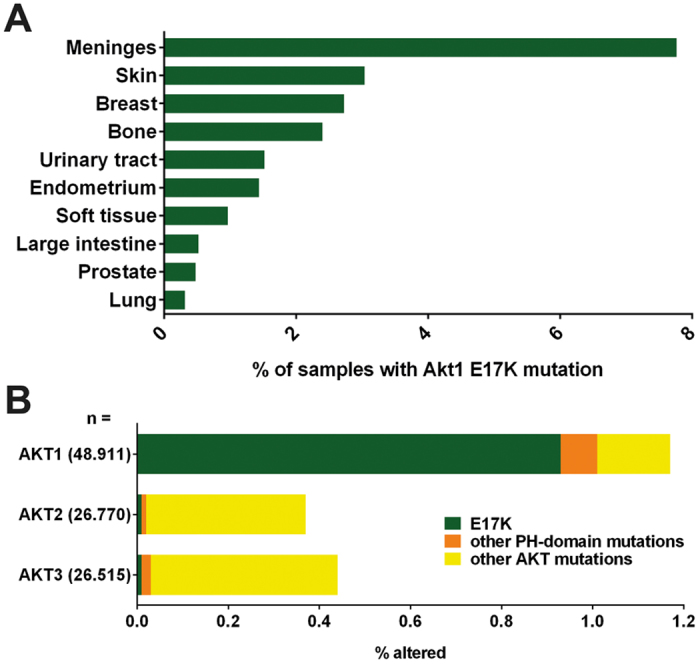
Akt1 is the prominent Akt isoform carrying the clinically relevant PH domain mutation E17K. (**A**) COSMIC Database analysis of the distribution of somatic Akt1-E17K mutations in tumour specimen of the respective tissues analysed; data represent the percentage of affected tissues of all tumour samples in the database in the respective tissue. (**B**) COSMIC Database analysis of the frequency of the PH-domain mutation E17K (green), other PH domain mutations (orange) or mutations in other regions (yellow) in the three Akt isoforms (Akt1, Akt2, Akt3) detected in tissue specimen of human cancer patients. Data represent percent of all samples with the respective isoform analysed.

The COSMIC database analysis of mutations in the three Akt isoforms found in cancer patients confirmed earlier reports about the occurrence of activating E17K mutations in the pleckstrin homology domain in various types of tumours – including frequently occurring tumours that are treated with radiotherapy such as skin, breast and prostate cancer (Fig. 1A). Interestingly, the E17K mutation was almost exclusively found in Akt1, occurring only at very low frequencies in Akt2 and Akt3 (Fig. 1B). In contrast, copy number alterations were more prominent in Akt2 and Akt3 when compared to gain of function mutations. Overexpression of the three Akt isoforms was observed in 2 to 18% of the tissue specimen analysed (Suppl. Fig. 1). Here, overexpression of Akt2 was predominant in tissue samples of endometrial, urinary tract, large intestine, oesophageal and pancreatic tumours, whereas overexpression of Akt3 was prevailing in skin and lung tumours. Although Akt1 overexpression was prominent in soft tissue tumours, it was still lower than that of Akt2 or Akt3. In all other analysed tissue types, the frequency of Akt1 overexpression was below 10%. As a consequence of the predominant occurrence of the clinical relevant E17K mutation in the Akt1 isoform, we focused our investigations on genetic alterations of Akt1.

### Mutations enhancing membrane localization of Akt1 stimulate proliferation *in vitro* and *in vivo*

To study the impact of activation-associated Akt1 mutants on cell behaviour, we used retroviral gene transfer to generate murine Tramp-C1 prostate cancer cells (TrC1) and Akt1 knock-out (Akt1−/−) mouse embryonic fibroblasts (MEF) stably expressing specific Akt1-eGFP fusion proteins. The set of different Akt1-eGFP fusion proteins comprised wild type (WT) Akt1, constitutively active Akt1, dominant negative Akt1 variants, and the empty vector pBEC (Suppl. Table 1). We chose TrC1 prostate cancer cells as a model cell line without known genetic alterations in the PI3K/Akt pathway and thus normal endogenous Akt activity as well as Akt1−/− MEF cells without endogenous Akt1 or other alterations in the PI3K/Akt signalling that may influence the E17K effects. To allow comparison of data obtained in the various generated cell lines, we adjusted the expression level of Akt1-eGFP fusion proteins by cell sorting based on eGFP-intensity. We first analysed potential differences in the basal phosphorylation state of the Akt1-eGFP fusion proteins in these cell lines, as well as the endogenous Akt protein in the TrC1 derived cell lines by Western Blot using phosphorylation-specific antibodies against phospho-T308 and phospho-S473. While overexpression of Akt1-WT provoked only a slight increase in phosphorylation at T308, Akt1-E17K and myrAkt1 strongly increased T308 phosphorylation. Furthermore, overexpression of Akt1-E17K and myrAkt1 also increased phosphorylation at S473, along with a higher amount of phosphorylated fusion protein compared to endogenous Akt1 (Suppl. Fig. 2A) supporting the assumption that Akt1-E17K and myrAkt1 are constitutively activated Akt1 mutants^10^. The increased phosphorylation of Akt1 at T308 and S473 was associated with enhanced proliferation leading to significantly shorter doubling times for the Akt1-E17K and myrAkt1 expressing TrC1 cells (Suppl. Fig. 2B–D). Furthermore, tumours generated from Akt1-E17K expressing Akt1−/− MEFs grew faster compared to tumours generated from Akt1-WT MEFs (Suppl. Fig. 2E), thereby corroborating the transforming ability and growth promoting effects of Akt1-E17K^3^,^26^. Interestingly, the phospho-mimicking variant Akt1-TDSD failed to enhance TrC1 proliferation suggesting that membrane recruitment or localization of Akt1 is required to enhance growth and proliferation signals. As expected, the dominant negative Akt1 variants, Akt1-K179A and Akt1-R25C slightly decelerated proliferation, presumably due to reduced kinase activity or impaired membrane recruitment (Suppl. Fig. 2B–D).

### Akt1-E17K and TDSD mutations but not myrAkt1 enhance cell survival upon IR

**Figure 2:**
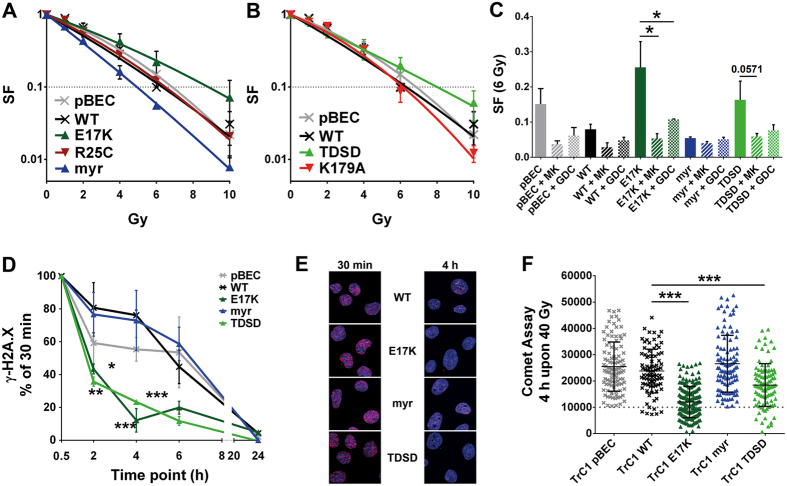
Akt1-E17K and TDSD mutations but not myrAkt1 enhance the repair of radiation-induced DNA DSBs and radiation resistance. (**A**,**B**) Effect of the various Akt1-mutants on long-term survival of irradiated TrC1 cells determined by standard colony formation assays upon irradiation with 0–10 Gy. (**C**) Effect of pre-treatment with 2 μM MK-2206 or GDC-0068 2 h prior to irradiation on long-term survival of TrC1 cells expressing the various Akt1-mutants exposed to 6 Gy as determined by colony formation assays. (**D**) TrC1 cells expressing the various Akt1-mutants were exposed to irradiation with 3 Gy and fixed at the indicated time points for immunofluorescence analysis. The kinetics of DNA DSB repair were followed by counting the amount of γ-H2A.X foci. (**E**) Representative photomicrographs of the γ-H2A.X assay with nuclei dyed in blue and phosphorylated H2A.X in magenta. TrC1 cells were fixed 30 min and 4 h upon 3 Gy. (**F**) TrC1 cells expressing the various Akt1-mutants were exposed to irradiation with 40 Gy. The amount of fragmented DNA as an indicator of DNA DSB repair was determined 4 h after irradiation by the neutral comet assay and quantification of the comet tail area. The dashed line demonstrates the mean background values of non-irradiated cells. Data show means ± SD (n = 3) or representative photomicrographs out of 3 independent experiments. *p < 0.05, **p < 0.01, ***p < 0.001; ANOVA test with Bonferroni correction.

To dissect the role of Akt1 in the cellular radiation response, we performed long-term and short-term survival assays with the generated cell lines. Overexpression of Akt1-WT resulted in a minor increase in Akt1 phosphorylation at S473 without and with irradiation and we detected only a minor protective effect on the survival of irradiated TrC1 Akt1-WT cells. Moreover, there was also a trend to improved clonogenic cell survival of pBEC-expressing TrC1 cells upon irradiation with 6 Gy when compared to Akt1-WT expressing TrC1 cell; however, this difference was not statistically significant (Fig. 2A, C). In contrast, the activation-associated Akt1 mutants Akt1-E17K and Akt1-TDSD significantly increased short-term and long-term survival of TrC1 cells exposed to IR (Fig. 2A, B; Suppl. Fig. 3B). Surprisingly, the constitutively active myrAkt1 radiosensitized TrC1 cells compared with Akt1-WT in long-term colony formation assays (Fig. 2A). The dominant negative Akt1-K179A mutation slightly decreased survival of irradiated TrC1 cells compared to Akt1-WT (Fig. 2B), whereas Akt1-R25C, which is thought to display reduced PIP_3_ coordination^24^ was ineffective (Fig. 2A).

Since Akt1 is known to impact expression and activity of cell cycle regulators^1^, we also analysed the effects of the Akt1-variants on cell cycle distribution by flow-cytometry-based determination of changes in the DNA content (Suppl. Fig. 3A). These investigations revealed that expression of the Akt1-variants did not significantly alter cell cycle distribution in exponentially growing non-irradiated TrC1 cultures. We also examined radiation-induced alterations (5 Gy) in cell cycle distribution in a similar approach at 24 hours after irradiation, but detected only subtle differences (Suppl. Fig. 3A). The only significant difference we observed was an increase of polyploid cells (>4n DNA-content) in irradiated myrAkt1-expressing cells. We speculate that this shift may be indicative for problems with proper cell division and increased genome instability. Moreover, the cells showed a similar arrest in the G2/M phase of the cell cycle upon irradiation. Of note, Akt1-E17K also increased radioresistance of TrC1 tumours *in vivo* when compared with Akt1-WT expressing TrC1 cells, as indicated by the apparent lack of growth retardation after a single high dose irradiation with 15 Gy (Suppl. Fig. 3C). To explore whether the observed radioresistance of TrC1 cells expressing Akt1-E17K or Akt1-TDSD can be reversed, we treated the cells either with the specific allosteric clinical phase II Akt-inhibitor MK-2206^29^,^30^, or the ATP-competitive Akt-inhibitor GDC-0068^31^ and found that both inhibitors radiosensitized Akt1-E17K and Akt1-TDSD expressing TrC1 cells to the levels of irradiated Akt1-WT TrC1 cells (Fig. 2C).

### Akt1-E17K and TDSD mutations but not myrAkt1 accelerate DSB repair

To gain further insight into the mechanisms of radiation response modulation by Akt1, we analysed the kinetics of induction and repair of DSB in our cell lines. For this purpose, we followed the kinetics of γ-H2A.X foci dissolving by immunofluorescence microscopy^32^. We normalized the values of γ-H2A.X-foci to the 30 min values of each cell line to emphasize the differences in the repair kinetics between the various cell lines.

Interestingly, expression of Akt1-E17K and Akt1-TDSD resulted in a faster resolution of γ-H2A.X foci in irradiated nuclei, whereas myrAkt1 tended to delay DSB repair (Fig. 2D, E). Thus, while in TrC1 cells expressing the Akt1-E17K or Akt1-TDSD mutants almost all γ-H2A.X foci were resolved within 2 to 4 hours after irradiation, about 80% of the initial damage persisted at these time points in TrC1 cells expressing either Akt1-WT or myrAkt1. The effect on DSB repair was even stronger in MEF Akt1−/− expressing the Akt1-E17K and Akt1-TDSD mutants. Moreover, we detected delayed DSB repair in MEF Akt1−/− expressing myrAkt1, or Akt1-K179A and Akt1-R25C compared with Akt1-WT expressing MEF Akt1−/− (Suppl. Fig. 4B). To corroborate our findings, we also monitored the effect of Akt1-mutants on DSB repair by measuring DNA fragmentation upon IR and its restoration using a neutral comet assay^33^. Importantly, the tail moment representing the level of DNA fragmentation was significantly reduced 4 hours after IR in Akt1-E17K and Akt1-TDSD, compared with Akt1-WT expressing TrC1 cells (Fig. 2F; Suppl. Fig. 4A) or MEF Akt1−/− (Suppl. Fig. 4C, D). Despite comparable levels of initial DNA damage at 30 min upon irradiation in all cell lines (Suppl. Fig. 4A, C), myrAkt1 and Akt1-WT cells displayed higher levels of residual DNA damage at 4 h, suggesting less efficient repair of DSBs (Fig. 2F; Suppl. Fig. 4D). To determine whether the accelerated kinetics of DSB repair in the Akt1-E17K and Akt1-TDSD mutants were linked to Akt1-activity, we incubated cells with MK-2206 or GDC-0068 and measured the kinetics of γ-H2A.X foci resolution. Interestingly, both Akt inhibitors compromised DSB repair promoting effects of Akt1-E17K in TrC1 cells (Suppl. Fig. 5B), thereby supporting observations concerning cell survival level (Fig. 2C; Suppl. Fig. 5A). These data demonstrate that the clinically relevant Akt1-E17K and the phospho-mimicking Akt1-TDSD mutant accelerate the repair of radiation-induced DSBs in TrC1 cells and that Akt inhibitors can overcome this effect.

### Nuclear localization of activation-associated Akt1-mutants is required for improved DSB repair

**Figure 3:**
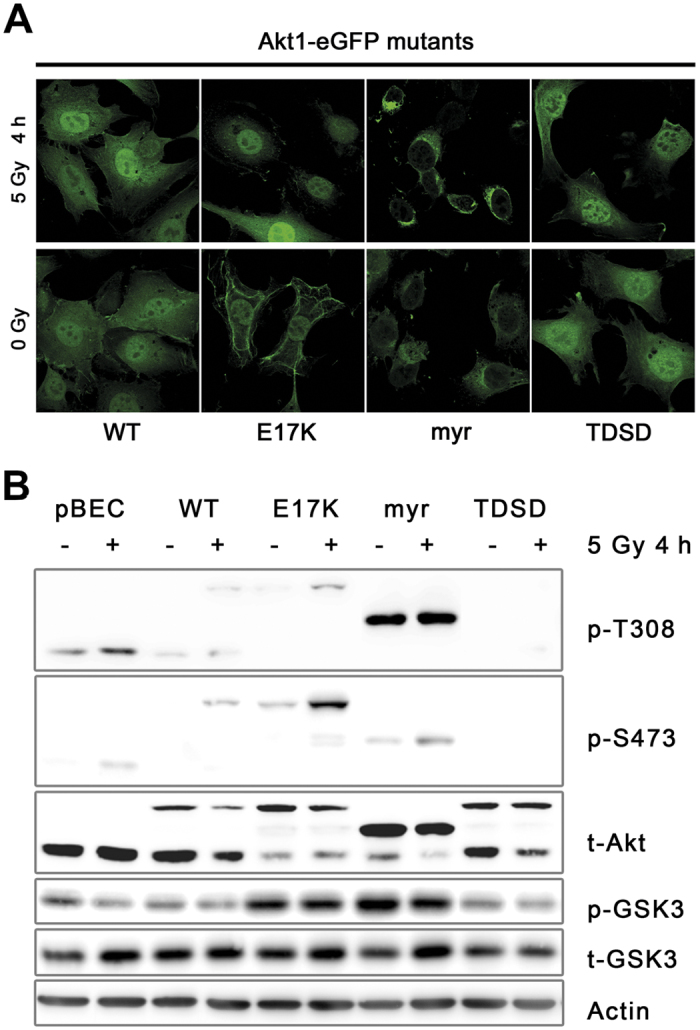
Akt1-E17K but not myrAkt1 shows pronounced nuclear localization and displays higher phosphorylation upon IR. (**A**) Subcellular localization of the eGFP-coupled Akt1 variants Akt1-WT, Akt1-E17K, myrAkt1 and Akt1-TDSD was determined in TrC1 cells by immunofluorescence of cells fixed in 3% PFA and permeabilized with 0.2% Triton X-100 in PBS without or 4 h after irradiation with 5 Gy. Data show representative photomicrographs out of 3 independent experiments. (**B**) Phosphorylation at T308 (p-T308) and S473 (p-S473) of endogenous Akt (60 kDa), myrAkt1 (70 kDa), and Akt1-mutants (87 kDa) was determined in cell lysates collected under conditions of exponential growth and 4 h after irradiation with 5 Gy. The total amount of Akt is demonstrated by t-Akt. Downstream Akt activity is shown by the phosphorylation of the Akt target protein GSK3 compared to the total amount of GSK3 (p-GSK3, t-GSK3). Data show representative cropped blots out of 3 independent experiments. Akt1-TDSD was neither recognized by the anti-pS473 nor by the anti-pT308 antibody because of the altered sequence.

As there were striking differences in the effects of the myrAkt1 mutant versus the Akt1-E17K or Akt1-TDSD mutants on DSB repair and radiosensitivity, we inquired whether there are links between these effects and Akt1 localization. We reasoned that irreversible binding of myrAkt1 to the cellular membranes, caused by the replacement of the PH-domain by a myristoyl anchor^34^, traps the protein in this cellular compartment and precludes essential nuclear functions (Fig. 3A). Fluorescence microscopy analysis revealed that Akt1-WT and Akt1-TDSD were ubiquitously present in non-irradiated and irradiated TrC1 cells within the cytoplasm as well as in the cell nucleus whereas myrAkt1 was predominantly located in vesicle-like cytoplasmic structures, presumably the endoplasmic reticulum and the Golgi apparatus, and did not show a more pronounced nuclear localization after irradiation. In contrast, Akt1-E17K was predominantly localized at the cytoplasmic membrane and in the nucleus in non-irradiated cells. Membrane localization of Akt1-E17K was reduced and nuclear localization increased at 4 h post-irradiation compared with non-irradiated control cells. These observations were corroborated by a relative quantification of green fluorescence intensity in the nucleus compared to the whole cell by an ImageJ-based method^35^: The relative fluorescence intensity in the nuclei of myrAkt1-eGFP expressing TrC1 cells was always significantly lower than WT controls (61% ± 8.1 lower intensity). We speculate that residual fluorescence detected in the nuclei of myrAkt1 expressing cells was mainly caused by the methodology issues e.g. scattered fluorescence from perinuclear regions. These findings support our assumption that only active Akt1 present in the nucleus is able to accelerate DNA repair and increase radiosensitivity, respectively.

Since endogenous Akt may exert a compensatory effect on DNA repair upon nuclear translocation at least in cells expressing pBEC or myrAkt1 that lack nuclear translocation of the transduced Akt1 variants, we also compared the levels of endogenous Akt1 and its phosphorylation state in the various TrC1 cell variants. Unexpectedly, overexpression of myrAkt1 and Akt1-E17K led to a decrease in levels of endogenous Akt when compared to Akt1-WT and pBEC cells. Furthermore, myrAkt1 and Akt1-E17K proteins displayed a higher basal phosphorylation level compared to the endogenous Akt, suggesting that in these cells the Akt-variants are the preferred target for activation of the signalling cascade when compared to endogenous Akt (Fig. 3B). We assume that the increased basal phosphorylation of the myrAkt1 and Akt1-E17K proteins is due to improved ability of myrAkt1 and Akt1-E17K to translocate to cellular membranes where activation of the cytoplasmic Akt takes place. These investigations also revealed that pBEC and Akt1-WT TrC1 cells differ in the levels of radiation-induced phosphorylation of endogenous Akt: while pBEC cells displayed increased phosphorylation of the endogenous Akt, Akt1-WT, Akt1-E17K and myrAkt1 cells mainly underwent phosphorylation of the Akt1-eGFP fusion protein (Fig. 3B), presumably because of the higher levels of the fusion protein compared to the endogenous protein levels.

### DNA-PKcs phosphorylates Akt1 at S473 upon DNA damage

**Figure 4:**
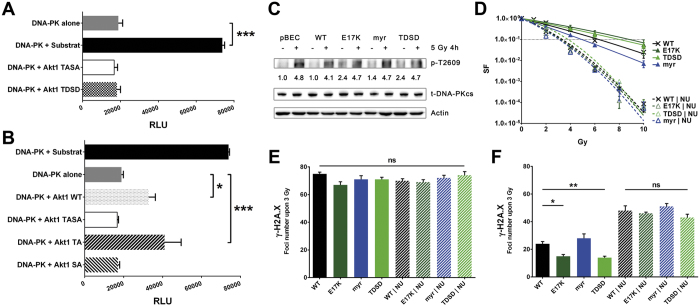
DNA-PKcs phosphorylates Akt1 at S473 upon DNA damage and is required for radiation resistance mediated by Akt1-TDSD and Akt1-E17K. (**A**) Purified Akt1 proteins of various Akt1 phospho-site mutants were used to test the phosphorylation hierarchy between DNA-PKcs and Akt1 in an *in vitro* kinase assay via a Luciferase-based Kinase assay measuring the ATP consumption. Data depict RLU – relative light units representing the emitted light produced by the Luciferase indicative for phosphorylation in the presence of DNA-PKcs and damaged DNA. Phosphorylation occurs only in the presence of a DNA-PKcs substrate but not in the presence of the constitutively active Akt1-TDSD. (**B**) Only Akt1-WT and Akt1-TA but not Akt1-SA and Akt1-TASA function as DNA-PKcs substrates. (**C**) Phosphorylation at T2609 (p-T2609) of DNA-PKcs (450 kDa) was determined in cell lysates collected under conditions of exponential growth and 4 h after irradiation with 5 Gy. The total amount of DNA-PKcs (t-DNA-PKcs) was used to normalize the level of its phosphorylation at T2609. The numbers demonstrate the fold-change of T2609 phosphorylation compared to non-irradiated Akt1-WT expressing TrC1 cells. Data show representative cropped blots out of 3 independent experiments. (**D**) The effect of pre-treatment with 2.5 μM NU7441 on the beneficial and adverse effects of the active Akt1-mutants on long-term survival of irradiated TrC1 cells (0–10 Gy) was analysed by standard colony formation assays. (**E**, **F**) The effect of pre-treatment with 2.5 μM NU7441 on the effects of the active Akt1-mutants on the kinetics of DNA DSB repair in irradiated TrC1 cells was analysed by γ-H2A.X assays. Data represent the amount of foci 30 min and 4 h upon 3 Gy. Data show means ± SD (n = 3). *p < 0.05, **p < 0.01, ***p < 0.001; ANOVA test with Bonferroni correction.

The phosphorylation hierarchy between Akt1 and DNA-PKcs in cells sustaining DNA damage remains controversial^16^,^20^,^36^. Therefore we developed a cell-free *in vitro* kinase assay to investigate the interactions and phosphorylation hierarchy between DNA-PKcs and Akt1 (Fig. 4A, B; Suppl. Fig. 6B). For this purpose we used the Akt1 phospho-mimicking mutant Akt1-TDSD, as well as mutants with impaired phosphorylation as a result of mutations to alanine of T308 and/or S473 - Akt1-TA, Akt1-SA and Akt1-TASA. The ability of Akt1-TDSD to phosphorylate Akt target proteins in solution was shown by an *in vitro* kinase assay, demonstrating a high kinase activity of purified Akt1-TDSD towards a GSK3 peptide (Suppl. Fig. 6A). Notably, active Akt1-TDSD failed to phosphorylate DNA-PKcs under the same conditions (Fig. 4A). In contrast, DNA-PKcs strongly phosphorylated Akt1 on S473 and this phosphorylation activity was eliminated when the Akt1-SA or Akt1-TASA mutants were used as substrates (Fig. 4B). We conclude that DNA-PKcs phosphorylates Akt1 at S473 in the presence of DNA DSBs, and postulate regulating interactions between DNA-PKcs and Akt1, possibly affecting D-NHEJ, in a cascade placing DNA-PKcs upstream of Akt. To examine potential indirect effects of active Akt1 mutants on the activation state of DNA-PKcs, we analysed whether the expression of the activation-associated Akt1 mutants affects the basal or radiation-induced phosphorylation of DNA-PKcs at T2609, the main autophosphorylation site of DNA-PKcs linked to D-NHEJ^37^. For this, we performed Western Blot analysis of lysates collected from exponentially growing intact cells without or with exposure to ionizing radiation (Fig. 4C). Interestingly, expression of Akt1-E17K and Akt1-TDSD increased the basal levels of DNA-PKcs phosphorylation at T2609 in our TrC1 cells compared to Akt1-WT expressing cells without irradiation whereas phospho-DNA-PKcs levels upon irradiation were comparable in all mutants (Fig. 4C).

### Inhibition of DNA-PKcs abolishes the radioprotective effect and the acceleration of DSB repair in Akt1-E17K and Akt1-TDSD expressing cells

Having shown that DNA-PKcs phosphorylates Akt1 at S473 in the presence of DSB, we investigated whether inhibition of DNA-PKcs compromises DSB repair and survival benefits in cells with hyperactive Akt1 mutants - as would be predicted when assuming mechanistic interconnections between the two kinases. For this purpose, we measured survival in the presence and absence of the DNA-PKcs inhibitor NU7441^38^, using colony formation as an endpoint. While expression of Akt1-E17K or Akt1-TDSD clearly reduced cell killing in the corresponding TrC1 cell lines, this effect was abrogated by pre-treatment with NU7441 (Fig. 4D). To explore the molecular basis of DNA-PKcs inhibition on Akt1-E17K and Akt1-TDSD induced radioresistance, we analysed the effects of NU7441 on the kinetics of DSB repair in these mutants. Once again, NU7441 abrogated the acceleration of DSB repair observed in Akt1-E17K and Akt1-TDSD expressing cells (Fig. 4E, F).

## Discussion

Intrinsic resistance to genotoxic therapies is a major obstacle to successful cancer therapy. Here, we provide evidence that two activating Akt1-mutations including the clinically relevant Akt1-E17K naturally occurring in human tumours and the artificial Akt1-TDSD promote resistance to ionizing radiation. Both mutants accelerated the repair of DNA DSB in TrC1 prostate cancer cells with low level of endogenous Akt1 activity as well as in Akt1-deficient MEFs. In contrast, the constitutively active myrAkt1 had the opposite effect. The resistance-promoting effect of Akt1-E17K and Akt1-TDSD was associated with the ability of Akt1-E17K and Akt1-TDSD to translocate to the nucleus, while myrAkt1 remained primarily at membrane structures. Mechanistically, *in vitro* kinase assays revealed that DNA-PK phosphorylates Akt1 at S473 and that Akt1-E17K and Akt1-TDSD-induced radiation resistance depends on DNA-PK. Importantly, the resistance-promoting effect of Akt1-E17K could be abrogated by pharmacologic inhibition of DNA-PK or Akt.

We demonstrate by *in silico* analysis that Akt1 is the dominant isoform harbouring gain-of-function mutations, particularly the PH domain mutation E17K, in tumour samples, whereas Akt2 and Akt3 undergo copy number variations with higher frequency (Fig. 1A). Consequently, we focused our study on Akt1 mutants. Although both, Akt1-E17K and myrAkt1, that are associated with increased localization to cellular membranes^3^,^34^, stimulate proliferation (Suppl. Fig. 2A–C). Only Akt1-E17K enhanced cell survival upon IR, whereas myrAkt1 had the opposite effect. However, similar to Akt1-E17K Akt1-TDSD, the other activation-associated Akt1 mutant with prominent nuclear localization (Fig. 3A), expedited the dissolving of γ-H2A.X foci and DNA DSB repair, and increased radioresistance (Fig. 2A, B, D). Importantly, the effects of active Akt1 mutants on DSB repair and cell survival could be abrogated by treating the cells with two different clinically relevant Akt inhibitors (Suppl. Fig. 5A, B) corroborating the functional relevance of Akt1 activity for the above findings.

Furthermore, our results demonstrate that the ability of the mutants to gain nuclear access is critical for the resistance-promoting effects of the Akt1 mutants. However, the observation that the highly active myrAkt1 enhances sensitivity to ionizing radiation highlights that cytosolic target proteins of Akt1 also affect the regulation of cell survival upon irradiation. The effects on radiosensitivity were not due to differences in cell cycle distribution of TrC1 cells expressing the different Akt1 variants at the time of irradiation, although the slightly increased fraction of myrAkt1 cells with intermediate DNA content (S phase cells) may be indicative of accelerated proliferation driven by myrAkt1. Moreover, a comparable fraction of the cells underwent an arrest in the G2/M phase of the cell cycle upon irradiation (Suppl. Fig. 3A). But myrAkt1 cells showed a higher fraction of polyploid cells at 3 days after irradiation. This observation suggests that proliferation-associated replication stress and mitotic failure result in increased genomic instability and may explain the increased radiosensitivity of myrAkt1 cells in long-term colony formation assays (Fig. 2A; Suppl. Fig. 3A, upper right panel). Although it was expected that pBEC cells and myrAkt1 cells should express similar levels of endogenous Akt1 that may exert a similar compensatory effect on DNA repair and radiosensitivity by its ability for nuclear translocation, we found that myrAkt1 and Akt1-E17K expressing TrC1 cells displayed lower levels of endogenous Akt that remained hypo-activated when compared to pBEC cells (Fig. 3B). Both effects will reduce the contribution of endogenous Akt to the observed phenotype in these cells. Furthermore, we postulate that in the myrAkt1 TrC1 cells the highly expressed myrAkt1 protein exerts a dominant effect over the remaining low levels of endogenous Akt and that a potential (small) repair-promoting effect of endogenous Akt1 will not be sufficient to override the dominant effect of myrAkt1 on cytoplasmic target proteins promoting cell proliferation and genomic-instability. In contrast, the endogenous Akt will play a more important role in pBEC cells and may even support the repair of radiation-induced DNA damage to some extent upon nuclear translocation. Such an effect may also contribute to the observed differences in DNA-repair capacity and radiosensitivity between myrAkt1 cells and pBEC cells. Unexpectedly, although Akt1-WT also displayed a prominent nuclear localization it did not improve the repair of radiation-induced DSB. But non-irradiated Akt1-E17K cells already displayed higher levels of T308 and S473 phosphorylation and phosphorylation at S473 further increased more prominently after IR compared to Akt1-WT (Fig. 3B). Thus, our findings strongly suggest that the activation state rather than the levels of nuclear Akt1 are crucial for Akt1-mediated radioresistance. This assumption is supported by a recent study in a murine glioma model showing that overexpression of Akt1 WT failed to significantly modulate the expression of proteins associated with DNA DSB repair and radiosensitivity^4^.

Interestingly, Akt1-E17K and Akt1-TDSD accelerated particularly the early phase of DSB repair, hinting to a role of active nuclear Akt1 in the fast component of DSB repair thought to reflect D-NHEJ, which is in agreement with other results^15^,^16^,^17^. In a mechanistic approach we therefore also explored the importance of DNA-PKcs in Akt1 mediated radioresistance. With our *in vitro* kinase assay we demonstrate that in presence of DNA DSBs DNA-PKcs specifically targets the S473 phosphorylation site and does not phosphorylate Akt at the T308 (Fig. 4A, B). The DNA DSBs were simulated by restriction endonuclease-introduced DSB into calf thymus double-stranded DNA in the activation buffer. These findings support regulating interactions between DNA-PKcs and Akt1 and place DNA-PKcs upstream of Akt1 in the phosphorylation hierarchy. Thereby, our results corroborate earlier *in vitro* reports as well as data from the *in silico* analysis of consensus sequences for DNA-PKcs and Akt1 phosphorylation sites^20^,^36^. However, we found a higher basal phosphorylation level of DNA-PKcs at T2609 in the TrC1 cells expressing the activation-associated Akt mutants Akt1-E17K or Akt1-TDSD but not myrAkt1 when compared to Akt1-WT expressing cells (Fig. 4C). This suggests that active Akt1 mutants with increased translocation to the nucleus can indirectly increase the activation state of DNA-PKcs. Since phosphorylation of DNA-PKcs at T2609 is mainly related to its activity in D-NHEJ activity^37^, these observations may provide an explanation for the accelerated DNA repair in these cell lines. Since the active Akt1-TDSD mutant failed to directly activate DNA-PKcs in our cell-free assay, our findings hint to an indirect effect of active nuclear Akt1 on DNA-PKcs activation, as suggested by others^16^,^19^,^23^.

**Figure 5:**
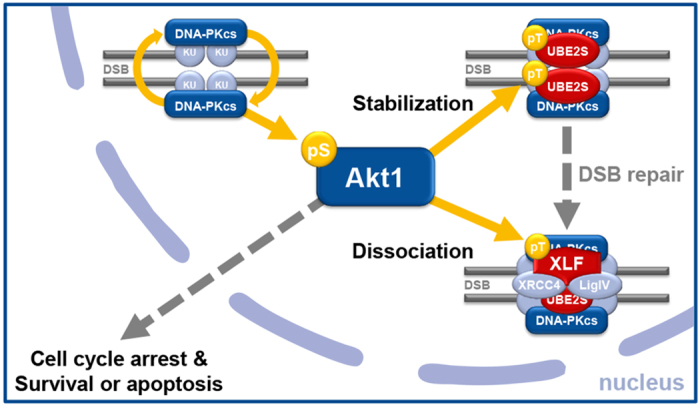
Proposed model of the interaction of Akt1 with the NHEJ pathway. Autophosphorylated active DNA-PKcs phosphorylates Akt1 at S473 at the DNA damage site. Activated Akt1 is now able to stabilize nearby D-NHEJ complexes via phosphorylation of UBE2S at its T152 leading to an enhanced association of UBE2S with Ku70 and the repair complex^39^. At a later stage of the repair Akt1 might introduce the dissociation of the D-NHEJ complex by phosphorylation of XLF at its phosphorylation site T181^17^. The phosphorylation of XLF triggers the dissociation of XLF from the DNA ligase IV/XRCC4 complex as a final step of the DSB repair. Furthermore, Akt1 functions as a switch upon genotoxic stress between cell cycle arrest/survival or apoptosis.

To corroborate our findings we next investigated whether inhibition of DNA-PKcs would abrogate the survival benefits of cells expressing the resistance-promoting Akt1 mutants - as would be predicted by the interaction between the two kinases. Indeed, inhibition of DNA-PKcs completely abrogated the beneficial effect of Akt1-E17K and Akt1-TDSD on DSB repair and survival of irradiated TrC1 cells (Fig. 4D–F), demonstrating that Akt1-induced protection from ionizing irradiation depends on DNA-PKcs. We speculate that activation of Akt1 by DNA-PKcs-dependent phosphorylation of Akt1 variants with enhanced nuclear access such as Akt1-E17K in the vicinity of radiation-induced DNA DSB has an indirect effect on D-NHEJ e.g. by increasing the dynamics of the formation and resolution of the corresponding DSB repair complexes. This assumption is supported by recent findings showing that activated Akt1 enhances D-NHEJ by stabilizing the repair complex via phosphorylation of UBE2S^39^. This effect was also observed in human glioma cells, where activated PI3K/Akt and ERK signalling led to enhanced DSB repair and inhibition of this pathway resulted in impaired repair^4^,^40^,^41^. A schematic representation of our hypothesis on the interaction of Akt1 with DNA-PKcs and proteins involved in DNA repair including these new findings is depicted in Fig. 5, showing in addition that facilitated activation of Akt1-E17K may enhance signalling of cell cycle arrest and apoptosis inhibition, as suggested by others^3^,^42^. Thereby, our findings substantiate the benefit of using PI3K/Akt pathway inhibitors to improve the efficacy of chemotherapy or radiotherapy and to overcome therapy resistance mediated by aberrant activation of Akt. But at the same time our findings also highlight the urgent need for the identification of critical downstream targets of (mutant) Akt1 in the DNA damage response that mediate radiation resistance^43^; the same holds true for altered signalling in cancer cells with copy number alterations of Akt2 or 3^4^. Potential candidates for this effect are the recently discovered Akt1 target proteins UBE2S^39^, XLF^17^ and MERIT40^14^.

Currently multiple clinical studies investigate the potential of PI3K/Akt pathway inhibitors to improve the outcome of chemotherapy and radiotherapy in cancer patients (phase 1 clinical studies involving BKM120 or BYL719 to radiosensitize cells)^44^,^45^,^46^. Although the Akt1-E17K mutation occurs in human cancer with only low frequency, our findings are of importance as they help to unravel the role of a cancer cell-specific mutation in the cellular radiation response and to understand the results of clinical studies testing the use of PI3K/Akt pathway inhibitors with distinct mechanisms of action in combination with chemotherapy or radiotherapy to overcome Akt1-mediated therapy resistance. In particular, our observations highlight the need to consider the patient-specific genetic alteration causing aberrant Akt activity, as multiple reports emphasise the association of different mutations with distinct drug responses^3^,^29^,^47^,^48^,^49^. A detailed mechanistic understanding of cancer cell-specific PI3K/Akt pathway alterations in the cellular radiation response and DNA repair is needed to guide the selection of genotype-specific drugs in precision cancer therapy in the future for more effective therapeutic interventions in tumours harbouring PI3K/Akt pathway alterations.

Further studies shall reveal whether the expression of the Akt1-E17K mutation in human tumour cell lines or primary cancer cells from patient samples is associated with increased radioresistance. However, we are convinced that the stably transduced Akt mutants in murine cancer cells are good models for studying the effects of cancer cell-specific genetic alterations in Akt1 on radiosensitivity and that therefore our conclusions remain sound. Indeed, one advantage of our model system is that we have a common parental cell line that expresses the empty vector, Akt1-WT, or one specific Akt1-mutant. Thus, the expression of the transduced Akt1-variant is the only difference among them. This cannot be ensured for human tumours with Akt1-mutations for which ”controls” may be impossible to find.

In summary, we identified novel aspects of the role of Akt1 in the cellular radiation response and provide evidence that the Akt1-E17K mutant naturally occurring in human tumours protects cancer cells from radiation-induced DNA damage. The link between expression of Akt1-E17K, improved DSB repair, and increased radioresistance highlights a potential use of Akt1-E17K as a biomarker for cancer cell radioresistance. It also strongly advocates the use of Akt1-E17K specific inhibitors as single agents or in combination with chemotherapy or radiotherapy for precision cancer therapy of patients harbouring Akt1-E17K positive tumours. The various genetic lesions promoting aberrant activation of Akt1 in cancer patients and the availability of drugs with different mechanisms of action highlight the opportunity for a mechanism-based matching of patients and individualized treatment to effectively abrogate intrinsic tumour cell radioresistance.

## Methods

### Colony Formation Assay

Clonogenic cell survival was tested in response to radiotherapy with doses between 1 and 10 Gy. Exponentially grown cells were seeded in 6-well plates or tissue culture dishes. Cells were irradiated 24 h after seeding and further incubated under standard culturing conditions for up to 11 days. Pre-treatment with the inhibitors MK-2206, GDC-0068 and NU7441 diluted in culture medium was performed 2 h before irradiation. For determination of colony formation cells were fixed in 3.7% formaldehyde and 70% ethanol, stained with 0.05% Coomassie blue. Colonies of at least 50 cells were counted.

### Immunofluorescence

Cells were seeded on glass coverslips placed in 12-well plates and irradiated 24 h later with 3 or 5 Gy, respectively. Subsequently, cells were fixed and permeabilized in 3% PFA/0.2% Triton-X100 for 15 min and incubated in blocking solution including 2% goat serum overnight at 4 °C. DNA was stained with Hoechst33342 (Thermo Scientific, Waltham, MA, USA) (3,7 mg/l in PBS) for 15 min at RT. Coverslips were mounted onto glass slides with DAKO mounting medium (Dako NA Inc., Carpinteria, CA, USA). Images were analysed by a Zeiss Axiovert 200 fluorescence microscopy with ApoTome and the ZEN imaging software (Carl Zeiss, Goettingen, Germany). The localization of Akt1-eGFP fusion proteins were quantified with the Focinator software developed in our laboratory^35^.

### γ-H2A.X Assay

γ-H2A.X foci were stained for 1 h at room temperature (RT) with Alexa Fluor^®^ 647 mouse anti-H2A.X (pS139) (BD Biosciences, San Jose, CA, USA) diluted 1:100 in blocking solution. γ-H2A.X foci were analysed by fluorescence microscopy and counted with the Focinator software developed in our laboratory^35^.

### Neutral Comet Assay

Comet assay was performed using a modified protocol of Olive and Banath^33^. Glass slides were pre-coated with 1% agarose. Cells were seeded in a 6-well plate, irradiated 24 h later with 40 Gy and collected at the indicated time point by trypsinization. Cells were gently resuspended in 120 μl of 1% 2-Hydroxyethylagarose from Sigma-Aldrich (St. Louis, MO, USA), directly pipetted onto the coated glass slides and immediately covered by a cover slip. Neutral lysis was performed in N1 buffer (2% Sarkosyl, 0.5 M Na_2_EDTA, 0.5 mg/ml proteinase K (pH 8.0)) for 3 h at 37 °C 5% CO_2_ and stopped with N2 buffer (90 mM Tris buffer, 90 mM boric acid, 2 mM Na_2_EDTA (pH 8.5)) for 15 min. Electrophoresis was conducted at 15 V (0.6 V/cm) for 25 min. The comets were stained with 50 μg/ml propidium iodide. For analysis at least 50 comets were examined for their tail area using the software OpenComet^50^,^51^.

### Protein expression and purification

The Akt1-HALO fusion proteins were expressed and purified according to manufacturer’s instructions of the HaloTag^®^ Protein Purification System kit (Promega Corporation, Madison WI, USA). The transfection was performed using the Trans-IT LT1 Transfection Kit following the manufacturer’s manual (Mirus Bio LLC, Madison, WI, USA). Purified proteins were diluted to concentration of 1 mg/ml, evaluated via Western Blot, and stored in purification puffer at −80 °C.

### DNA-PK Assay

The kinase assays were performed in a 96-well plate with 4 μg of the purified Akt1-HaloTag proteins and 20 units of DNA-PK by using the ADP-Glo™ Kinase Assay and the DNA-PK Kinase Enzyme System (Promega Corporation, Madison WI, USA) according to manufacturer’s instructions. The experimental set-up is depicted in Suppl. Fig. 6B.

### Animal Studies

All animal experiments were conducted in accordance with the relevant guidelines and regulations postulated by the German law for care and use of laboratory animals. Experimental protocols were approved by the appropriate licensing Animal Protection Board (German State Agency for Nature, Environment and Consumer Protection) and performed in accordance with the respective approval (approval code Az. 84-02.04.2014.A244). Immunodeficient male NMRI (nu/nu) nude mice were purchased from the University Hospital Essen (age 6–12 weeks). Mice were bred and housed under specific pathogen-free conditions in the Laboratory Animal Facility of the University Hospitals Essen in an individually ventilated cage rack system (Techniplast, West Chester, PA, USA) and fed with sterile high-calorie laboratory food (Sniff, Soest, Germany). Xenograft tumours of Akt1 variants expressing cells were generated by a subcutaneous injection of 5 × 10^5^ cells in 50 μl PBS mixed with 50% Matrigel (growth factor reduced Matrigel Matrix, Corning, Tewksbury, MA, USA) into the right hind leg. Food and drinking water were available ad libitum. Injections of cells and measurements of the tumours were performed blinded to ensure a randomization and an independency of investigator-related bias. The keeping and sacrificing of the mice was carried out according to the guidelines of the GV-SOLAS. For radiation therapy mice were anesthetized (2% isoflurane) and tumours were exposed to a single dose of 5 Gy ± 5% in 5 mm tissue depth (∼1.53 Gy/min, 300 kV, filter: 0.5 mm Cu, 10 mA, focus distance: 60 cm) using a collimated beam (field size: 25 × 13 mm) with a XStrahl RS 320 cabinet irradiator (XStrahl Limited, Camberly, Surrey, Great Britain).

### Statistical Analysis

Data represent mean values of at least 3 independent experiments standard deviation (SD). Data analysis was performed by one-way or two-way ANOVA tests with Bonferroni two-pair comparison post-test. P values ≤ 0.05 were considered as significant. In case of normal distribution, t-tests were used to compare two data sets.

## Additional Information

**How to cite this article**: Oeck, S. *et al*. Activating Akt1 mutations alter DNA double strand break repair and radiosensitivity. *Sci. Rep.* **7**, 42700; doi: 10.1038/srep42700 (2017).

**Publisher's note:** Springer Nature remains neutral with regard to jurisdictional claims in published maps and institutional affiliations.

## Supplementary Material

Supplementary Information
