# Supplementary material for: Activating Akt1 mutations alter DNA double strand break repair and radiosensitivity
Source: Sci Rep. 2017 Feb 17;7:42700. doi: 10.1038/srep42700 (PMC5314324; doi:10.1038/srep42700)
Supplement: Supplementary Information [file srep42700-s1.pdf]

Supplementary Information

Activating Akt1 mutations alter DNA double strand break repair and radiosensitivity

Author list and affiliations:

Oeck S[1], Al-Refae K[1], Riffkin H[1], Wiel G[1], Handrick R[1,2], Klein D[1], Iliakis G[3], Jendrossek V[1]*

[1] Institute of Cell Biology (Cancer Research); University of Duisburg-Essen; Medical School; Virchowstrasse 173; 45122 Essen; Germany

[2] Institute of Applied Biotechnology; University of Applied Sciences Biberach; Hubertus-Liebrecht-Str. 35; 88400 Biberach; Germany

[3] Institute of Medical Radiation Biology; University of Duisburg-Essen; Medical School; Hufelandstr. 55; 45122 Essen; Germany

*** Correspondence to:**

Verena Jendrossek, Institute of Cell Biology (Cancer Research), Department of Molecular Cell Biology; University of Duisburg-Essen; Medical School; Virchowstrasse 173; 45122 Essen; Germany; Phone: 49-201-7233380; Fax: +49-201-7235904; E-mail: verena.jendrossek@uni-due.de

SI Methods

Reagents. The inhibitors MK-2206, GDC-0068 and NU7441 were obtained from SelleckChem (Houston, TX, USA). G418 was purchased from Biochrom AG (Berlin, Germany). All other chemicals were from Sigma-Aldrich (St. Louis, MO, USA) and Thermo Fisher Technology (Waltham, MA, USA) if not otherwise indicated.

Antibodies. The γ-H2A.X-AF647 antibody was obtained from Becton Dickinson (#560447, Franklin Lakes, NJ, USA). Mouse monoclonal ß-Actin antibody was obtained from Sigma-Aldrich (#A5441, St. Louis, MO, USA). All other antibodies were purchased from Cell Signaling Technology (Danvers, MA, USA): Akt (pan) #4685 (t-Akt), Phospho-Akt (Thr308) XP #13038 (p-T308), Phospho-Akt (Ser473) XP #4060 (p-S473), GSK-3β XP #12456 (t-GSK3), Phospho-GSK-3β XP #5558 (p-GSK3).

Cell Culture and Treatment. TRAMP-C1 murine prostatic adenocarcinoma cells were purchased from ATCC (Bethesda, MD, USA). Murine embryonic fibroblasts (MEF) from Akt wildtype (WT) and Akt1 knock-out (Akt1 -/-) mice were kindly provided by Morris J. Birnbaum (Philadelphia, PA, USA). Cells were cultured in DMEM (Thermo Fisher Technology, Waltham, MA, USA) medium supplemented with 10% (v/v) foetal calf serum (Biochrom AG, Berlin, Germany) and maintained in a humidified incubator (Labotect, Goettingen, Germany) at 37°C and 5% CO_2. All cell lines were validated and monitored using microscopy and Western Blots analysis. All cell lines have monthly been tested for mycoplasma contamination. Cells were transduced with viruses produced in Platinum-E cells (ATCC, Bethesda, MD, USA) using the pBABE-retroviral system according to Morgenstern and Morita [45,46]. The transduced cells were cultured two weeks before and after eGFP-based cell sorting in DMEM medium containing 10% FCS, 1x Pen/Strep, 1 µg/ml Ciprofloxacin and 4 µg/ml Puromycin for selection and prevention of contamination.

Irradiation. For radiation treatment cells were exposed 24 h after seeding to 1-10 Gy using an X-RAD 320 X-Ray Biological Irradiator with X-RAD MIR-324 x-ray tube (Precision X-Ray Inc, North Branford, CT, USA). Non-irradiated controls were handled in parallel but kept outside of the irradiator during treatment. Cell number and viability was quantified by counting cells with a CASY cell counter from Innovatis (Reutlingen, Germany).

Western Blot Analysis. Cell pellets were lysed for 30 min on ice in RIPA buffer (50 mM HEPES, pH7.5, 1% Triton X-100, 150 mM NaCl, 10 mM NaPP, 1 mM EDTA, 10 mM NaF, 2 mM Na_3VO_4, Protease Inhibitor Cocktail Tablets from Sigma-Aldrich (St. Louis, MO, USA) and lysed with ultrasound (Bandelin Electronic, Berlin, Germany). Lysates were subsequently centrifuged 10 min at 14.000 rpm at 4°C and the protein concentration of the supernatants was adjusted to an equal protein amount using the Bradford Protein Assay (BIO-RAD, Munich, Germany). The protein solutions were incubated with 4x sample buffer (300 mM TRIS-HCl pH 6.8, 2.4% SDS, 60% Glycerol, 30% ß-Mercaptoethanol, 3.6

mg/100ml bromphenol blue) for 10 min at 95°C. For Western blot analysis proteins were separated by SDS-PAGE and blotted onto PVDF-membranes (Roth, Karlsruhe, Germany). After blocking with 5% (w/v) non-fat dry milk, membranes were incubated at 4°C overnight with the respective primary antibody (1:20.000 for β-actin, 1:1000 for all other antibodies). Afterwards, the membranes were incubated for 1 h at RT with the secondary antibody (anti-IgG-HRP 1:2000) diluted in 5% (w/v) non-fat dry milk. Detection was performed by chemiluminescence imaging using ECL Prime Western blotting detection reagent (GE Healthcare, Pittsburgh, PA, USA), a FUSION Solo and the Fusion software (peqlab, Erlangen, Germany).

Quantification of cell viability and cell proliferation. The number of living cells was determined using CASY technology based on an electric field multi-channel cell counting system from Innovatis AG (Reutlingen, Germany). For this, cells were harvested by trypsinization, re-suspended in fresh medium, diluted with CASYton solution from Hoffmann-La Roche AG (Basel, Switzerland) and measured. Metabolic activity and cell viability was measured with the Wst-1 assay according to the manufacturer´s instructions (Roche Applied Science, Mannheim, Germany) by an ELISA reader (Bio-Tek, Bad Friedrichshall, Germany).

Flow cytometry. Flow cytometric analysis of the cell cycle was based on propidium iodide (PI) staining of the DNA in a hypotonic citrate buffer according to the Nicoletti protocol [49]. In brief, cells were resuspended in 200 µL of the staining solution (comprised of 0.05% Triton X-100, 50 µg/mL propidium iodide and 0.1% sodium citrate in PBS), incubated in the dark for 30 min and measured with a BD Accuri C6 flow cytometer (BD Biosciences, San Jose, CA, USA) was performed. Flow cytometry analyses were performed in BD Accuri C6 Software (BD Biosciences, San Jose, CA, USA).

Supplemental Tab. 1: Akt1 constructs and effects of different variants

Variants	Type	Effect
**Akt1-WT**	Wild type	-
**Akt1-E17K**	Dominant active	Enhanced PIP_3 coordination
**Akt1-R25C**	Dominant negative	Impaired PIP_3 coordination
**Akt1-K179A**	Dominant negative	Impaired ATP coordination
**myrAkt1**	Constitutively active	No PH-domain, N-terminal myristoylation
**Akt1-T308D/S473D**	Constitutively active	T308/S473 phosphorylation mimicked

Supplemental figures

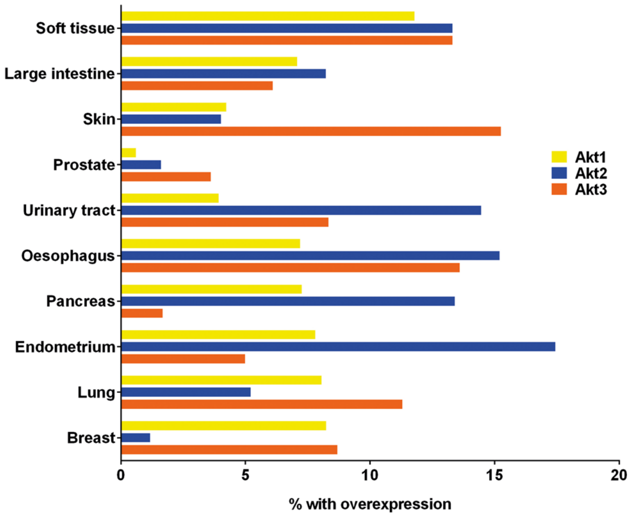

Supplemental Fig. 1: COSMIC database analysis of tumour tissues with enriched *Akt* expression. Percentage of tumour samples expressing at least more than 2-fold levels of the Akt1, Akt2 or Akt3 isoforms, respectively; data represent the percentage of all tumour samples of the respective tissue.

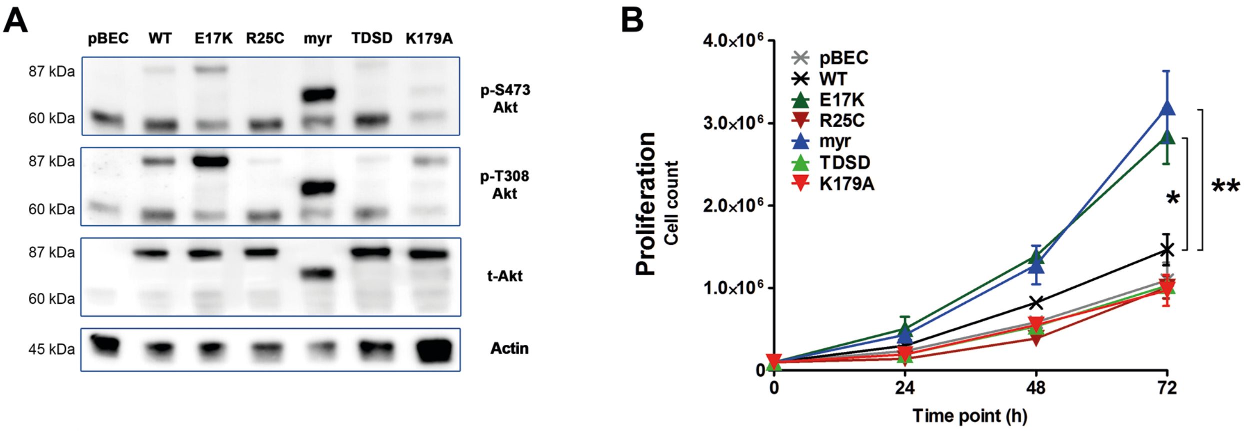

C

	Tramp-C1	pBEC	WT	myr	K179A	E17K	R25C	TDSD
Doubling time (h)	25.0	26.3	19.7	9.0	29.6	10.1	28.5	28.0

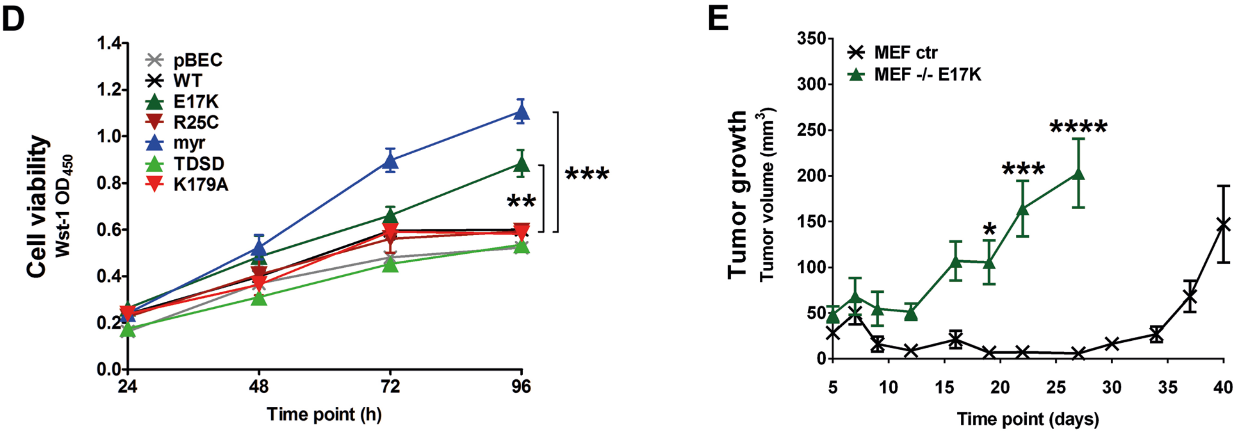

Supplemental Fig. 2: Akt1 mutations impact on Akt1-phosphorylation and cell proliferation. A Expression levels and phosphorylation at S473 and T308 of the various Akt1-mutations expressed in TrC1 cells were analysed by Western Blot. **B** The number of viable cells was determined at the indicated time points by cell counting using a CASY cell counter. Data show means ± SD (n=3). **C** Doubling times were calculated based on cell counting shown in **B**. **D** Determination of the number of viable cells at the indicated time points by using the Wst-1 assay. Data show means ± SEM (n=4). **E** Growth curves of tumours derived from Akt1-WT MEFs or MEF Akt1 -/- expressing Akt1-E17K in NMRI nude mice was followed by measuring the tumour volume twice weekly. Data show means ± SD (n=6 mice per group). * p<0.05, ** p<0.01, *** p<0.001, **** p<0.0001; ANOVA test with Bonferroni correction).

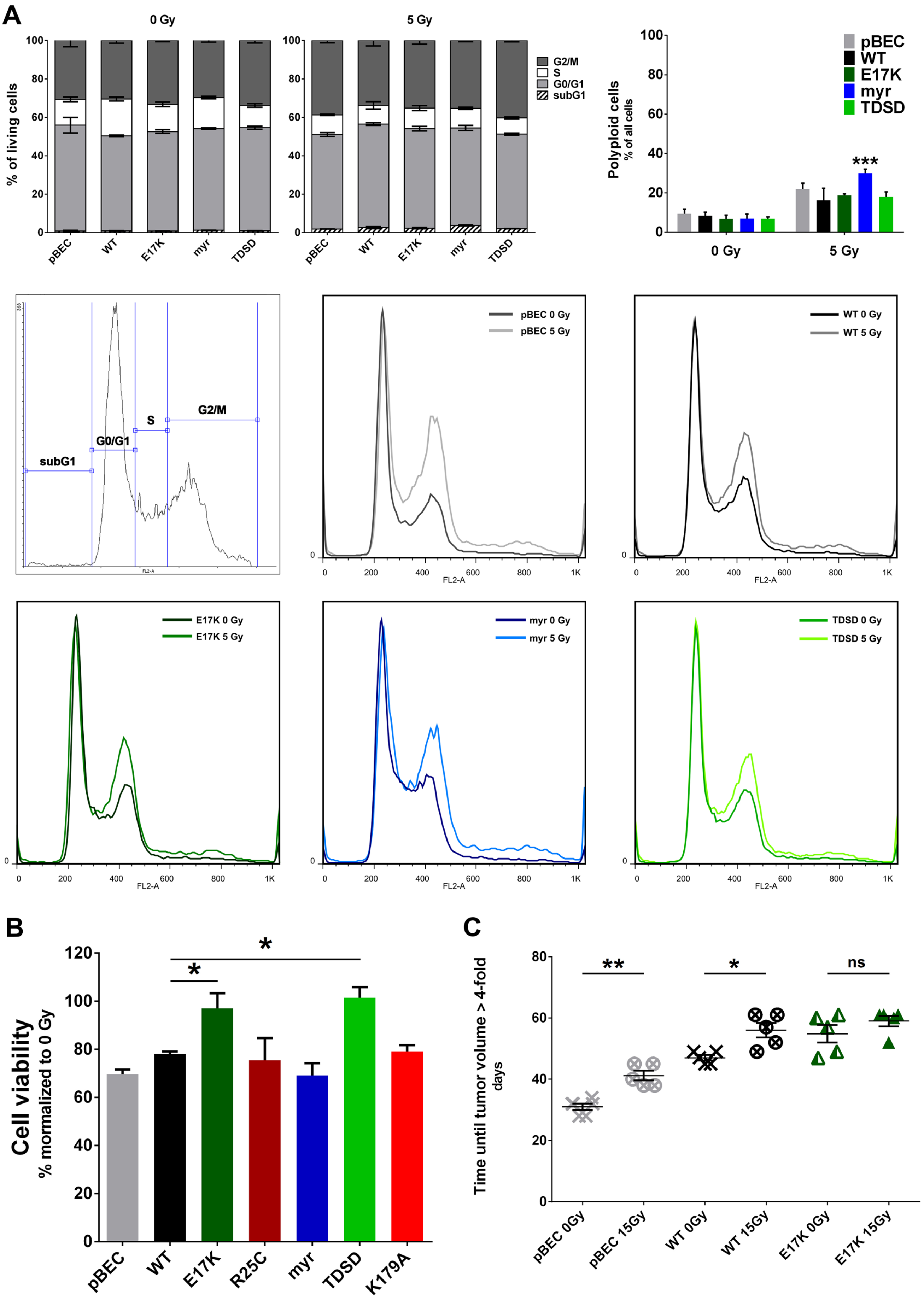
A
0 Gy
5 Gy
% of living cells
G2/M
S
G0/G1
subG1
pBEC
WT
E17K
myr
TDSD
Polyploid cells
% of all cells
***
subG1
G0/G1
S
G2/M
FL2-A
pBEC 0 Gy
pBEC 5 Gy
WT 0 Gy
WT 5 Gy
E17K 0 Gy
E17K 5 Gy
myr 0 Gy
myr 5 Gy
TDSD 0 Gy
TDSD 5 Gy
B
Cell viability
% mormalized to 0 Gy
R25C
K179A
C
Time until tumor volume > 4-fold
days
**
*
ns
pBEC 0Gy
pBEC 15Gy
WT 0Gy
WT 15Gy
E17K 0Gy
E17K 15Gy

1

Supplemental Fig. 3: Akt1-E17K mediates increased radiation resistance of TrC1 cells *in vitro* and *in vivo.* **A** Cell cycle analysis of the various Akt1-mutants was performed without radiation (upper left panel) and 24 h after a single dose of 5 Gy (upper middle panel). The percentages of subG1, G0/G1, S, G2/M TrC1 cells are displayed. Additionally, polyploid cells were analysed without IR and 72 upon 5 Gy (upper right panel). Data show means ± SD (n=3). *** p<0.001; ANOVA test with Bonferroni correction. The lower panels show the gating strategy and representative histograms. **B** Effect on short-term survival of TrC1 cells measured 72 h after exposure to 5 Gy using the Wst-1 assay. Data show means ± SD (n=3). * p<0.05, ** p<0.01; ANOVA test with Bonferroni correction. **C** Xenograft tumours of TrC1 cells expressing pBEC, Akt1-WT or Akt1-E17K were generated on NMRI nude mice and irradiated 15 days with 0 Gy (sham controls) or 15 Gy. Data show the time until xenograft tumours reached 4-fold volume. Data show means ± SD (n=6). * p<0.05, ** p<0.01, ns: not significant; ANOVA test with Bonferroni correction.

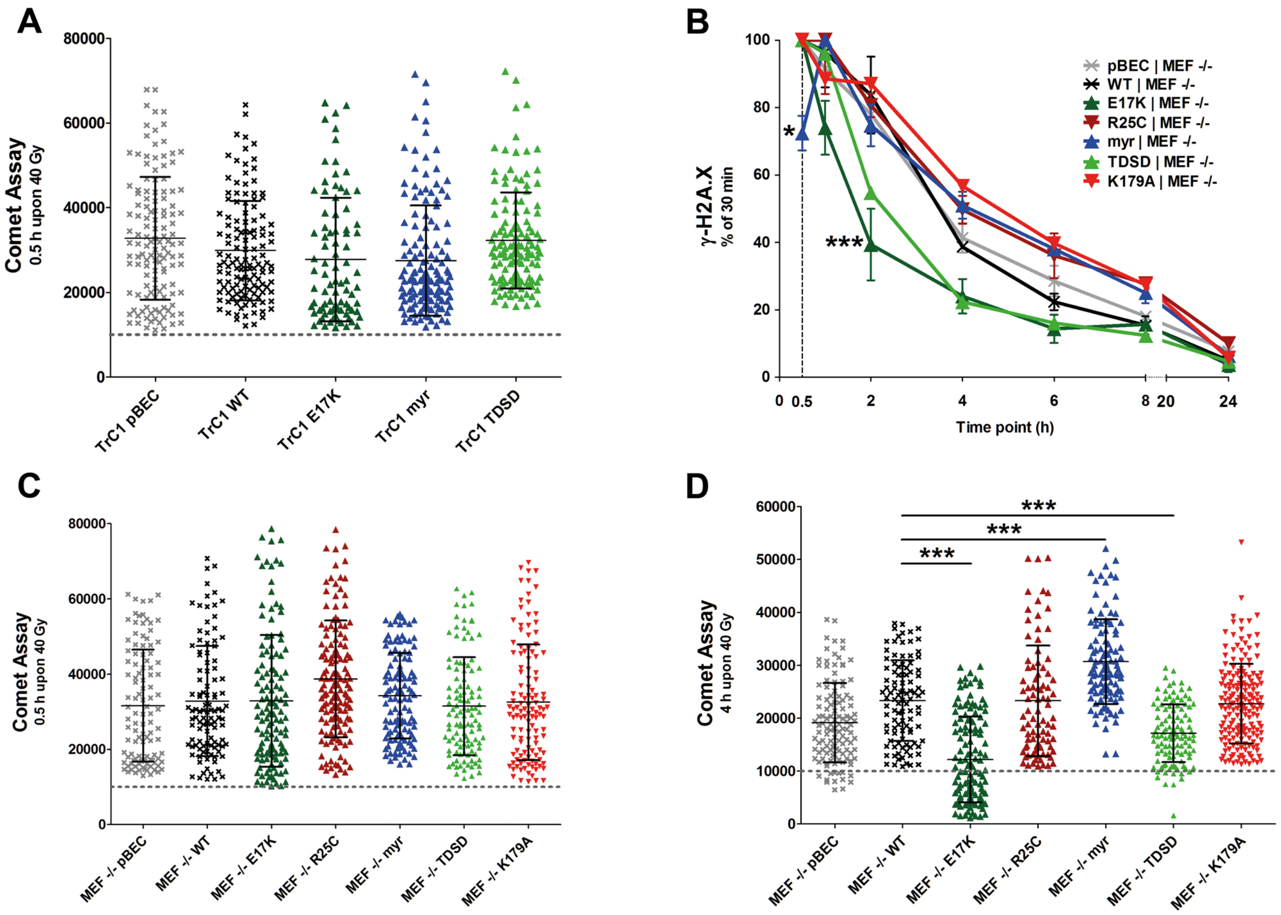

Supplemental Fig. 4: Active Akt1 mutants alter DNA DSB repair in TrC1 and MEF Akt1 -/- cells. A Determination of DNA fragmentation in TrC1 cells expressing pBEC or the various Akt1 mutants by neutral comet assay 30 min after irradiation with 40 Gy. **B** γ-H2A.X assay performed in MEF Akt1 -/- cells expressing the various Akt1 mutants at the indicated times on irradiation with 3 Gy. Determination of DNA fragmentation in MEF Akt1 -/- cells expressing the various Akt1-mutants by neutral comet assay performed 30 min (**C**) and 4 h (**D**) on irradiation with 40 Gy. Data show means ± SD (n=120). *** $p<0.001$; ANOVA test with Bonferroni correction.

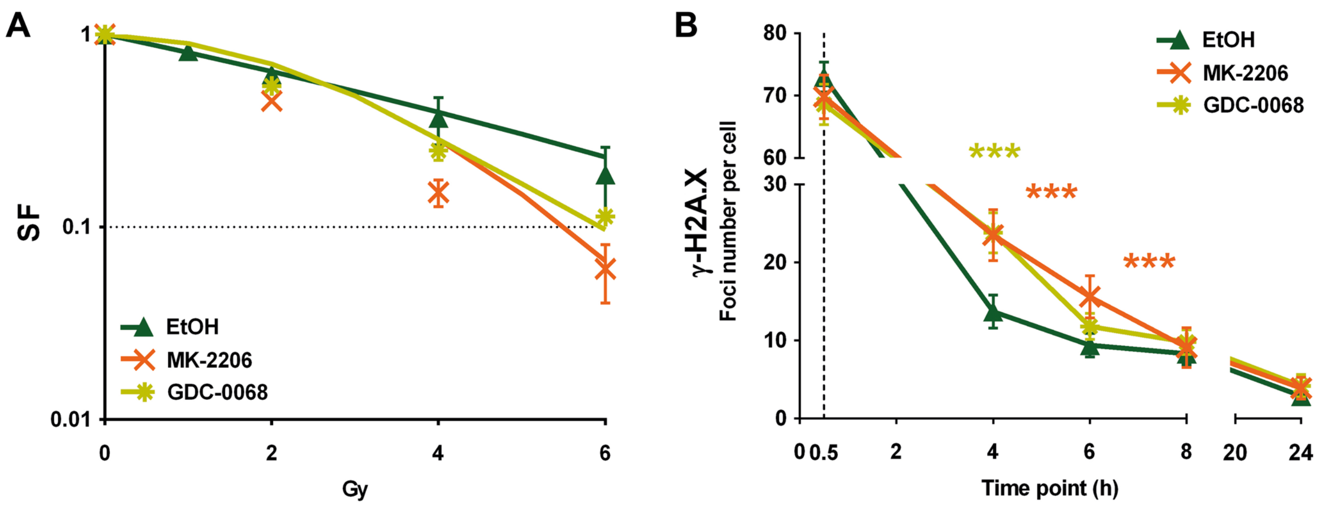

Supplemental Fig. 5: Clinically relevant Akt inhibitors negate the radioresistance and the DNA repair-promoting effect of Akt1-E17K. A The effect of pre-treatment with 2 µM MK-2206 and GDC-0068 on the resistance-promoting effect of Akt1-E17K in TrC1 cells as determined by colony formation assays upon irradiation (0-6 Gy). EtOH was used as a solvent control. Data show means ± SEM (n=3). **B** The effect of pre-treatment with 2 µM MK-2206 and GDC-0068 on the beneficial effects of the active Akt1-E17K on the kinetics of DNA DSB repair in irradiated TrC1 cells (3 Gy) was analysed by γ-H2A.X assay. The kinetics of DNA DSB repair were followed by counting the amount of γ-H2A.X foci. Data represent the amount of foci at the indicated time points. EtOH was used as a solvent control. Data show means ± SD (n=3). *** p<0.001; ANOVA test with Bonferroni correction.

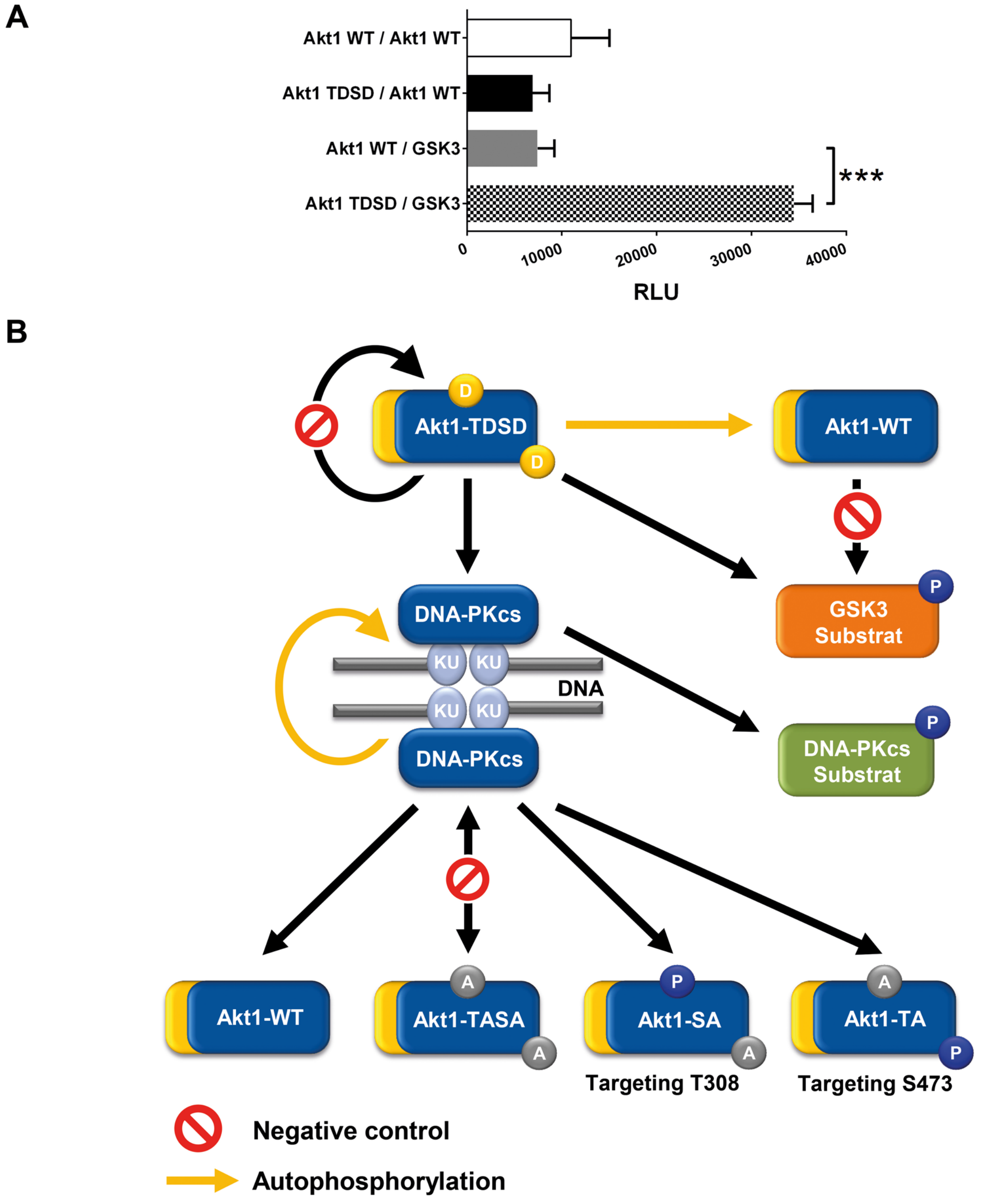

Supplemental Fig. 6: Determination of the phosphorylation hierarchy between Akt1 and DNA-PKcs by an *in vitro* kinase assay. A Kinase assay with Akt1-WT and TDSD variants with GSK3 target peptide performed to detect the kinase activity. Data show means ± SD (n=3). *** p<0.001; ANOVA test with Bonferroni correction. **B** Scheme of experimental set-up of the Akt1/DNA-PKcs kinase assay.

Supplemental Full Western Blots

p-T308 Akt1-eGFP (87 kDa)
p-T308 myrAkt1-eGFP (72 kDa)
p-T308 Akt endogenous (60 kDa)

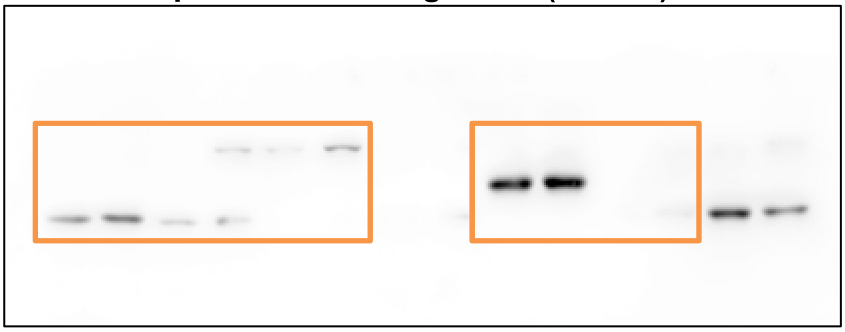

p-S473 Akt1-eGFP (87 kDa)
p-S473 myrAkt1-eGFP (72 kDa)
p-S473 Akt endogenous (60 kDa)

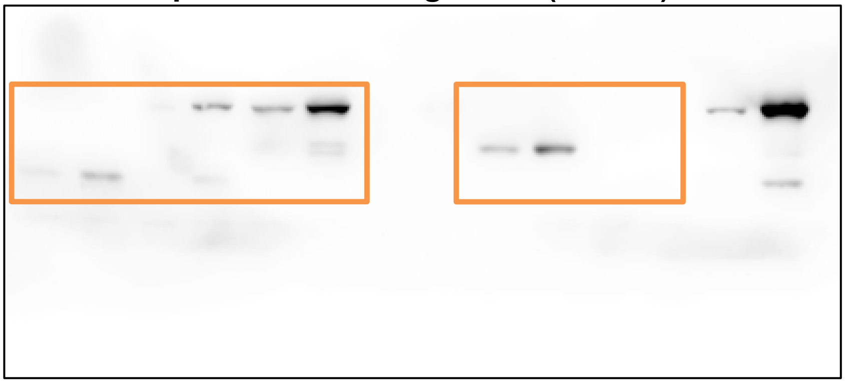

total Akt1-eGFP (87 kDa)
total myrAkt1-eGFP (72 kDa)
total Akt endogenous (60 kDa)

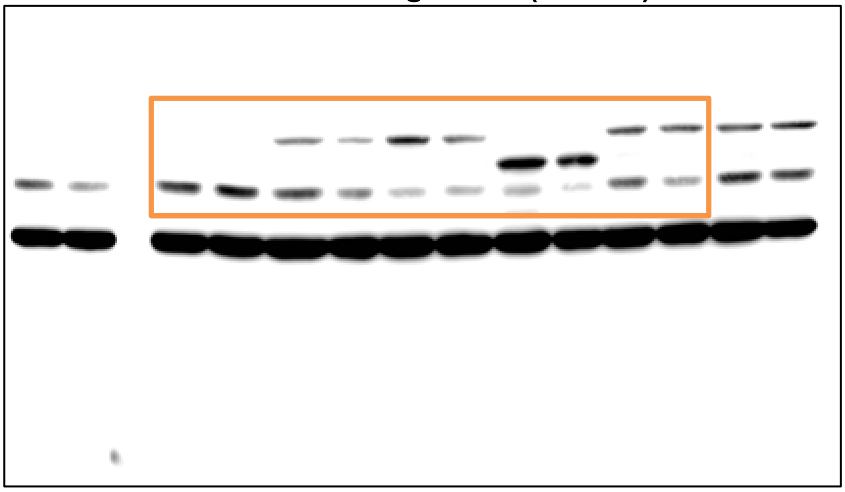

p-GSK3β (46 kDa)

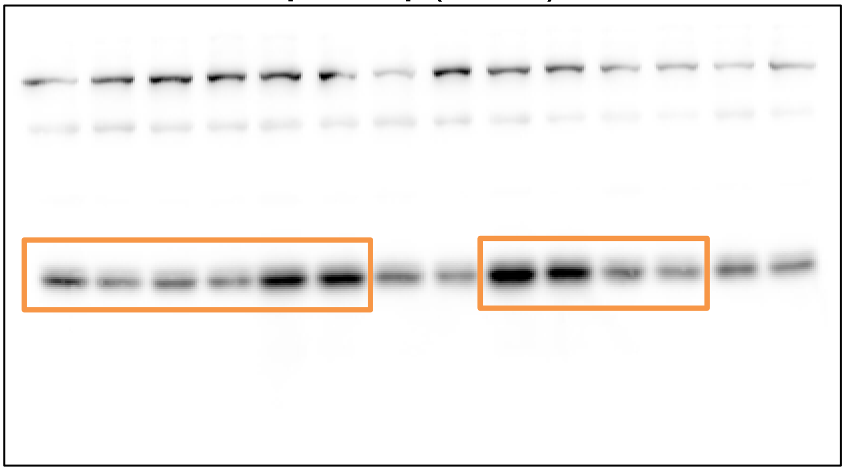

Total GSK3β (46 kDa)

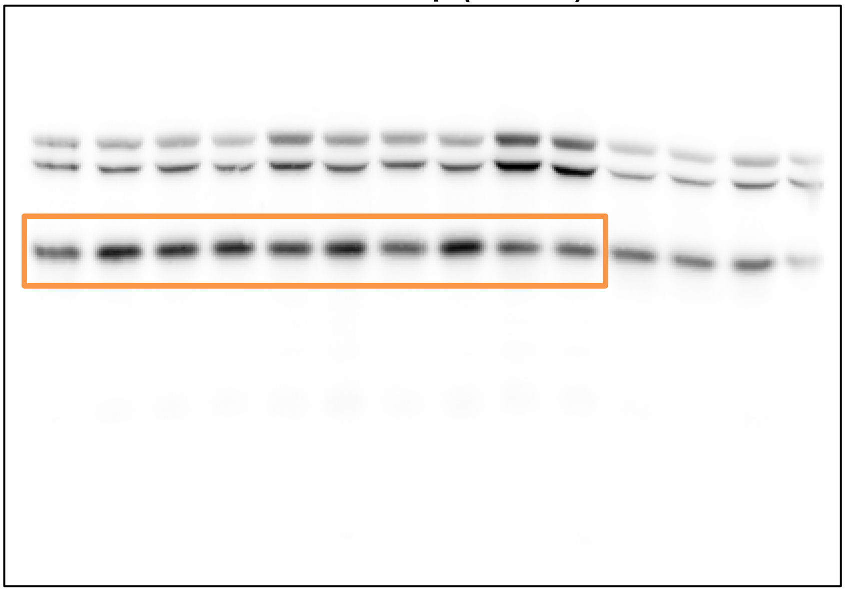

β-Actin (43 kDa)

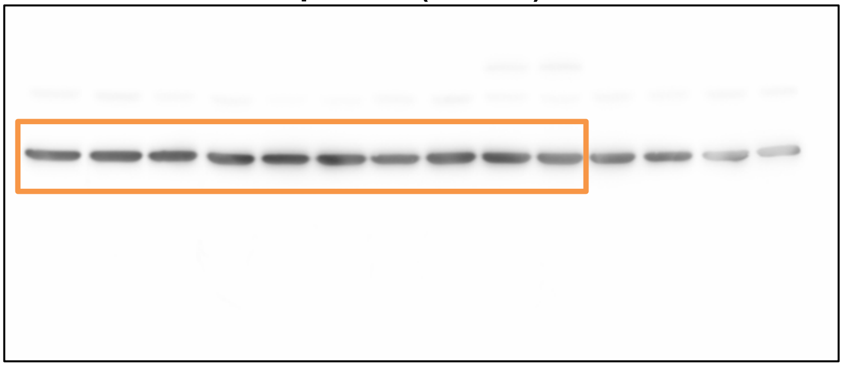

Full Western Blots related to Fig. 3B: Akt1-E17K but not myrAkt1 shows pronounced nuclear localization and displays higher phosphorylation upon IR. Indicated proteins were detected with protein-specific antibodies on Western Blots. Equal protein amounts (30 µg, whole cell lysate) were loaded. β-Actin was used as loading control.

p-DNA-PK (approx. 450 kDa)

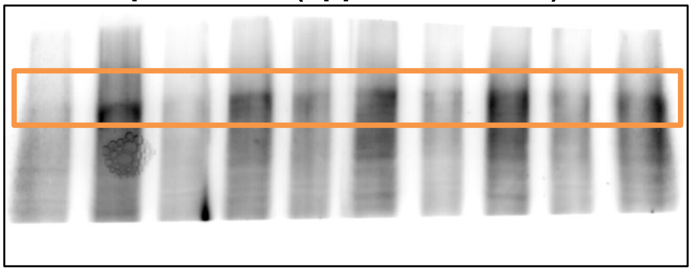

Total DNA-PK (approx. 450 kDa)

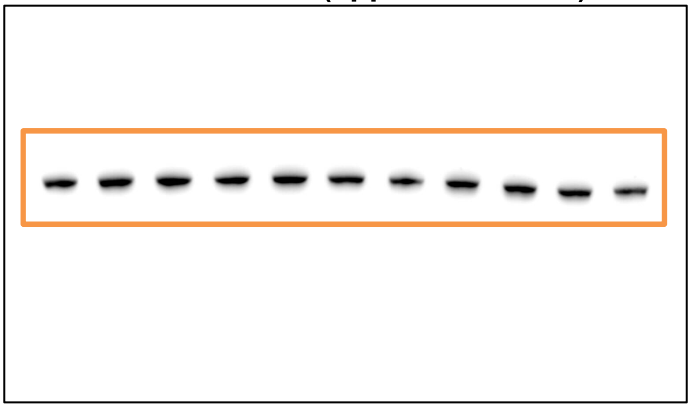

β-Actin (43 kDa)

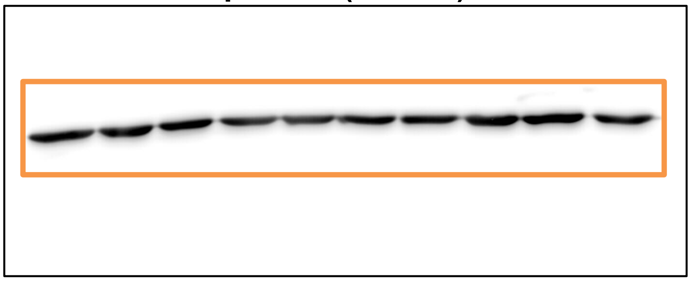

Full Western Blots related to Fig. 4C: DNA-PKcs phosphorylates Akt1 at S473 upon DNA damage and is required for radiation resistance mediated by Akt1-TDSD and Akt1-E17K. β-Actin (43 kDa), total DNA-PK (approx. 450 kDa), p-DNA-PK (approx. 450 kDa). Indicated proteins were detected with protein-specific antibodies on Western Blots. Equal protein amounts (30 µg, whole cell lysate) were loaded. p-DNA-PK levels were normalized to their related total DNA-PK signals. β-Actin was used as loading control.
